# Supplementary material for: The Effect of the Sterile Insect Technique on Vibrational Communication: The Case of Bagrada hilaris (Hemiptera: Pentatomidae)
Source: Insects. 2023 Apr 2;14(4):353. doi: 10.3390/insects14040353 (PMC10142036; doi:10.3390/insects14040353)
Supplement: Supplementary file 1 [file insects-14-00353-s001.zip › insects-2283990-supplementary.pdf]

Table S1. Akaike Criterion Information (AIC) of candidate models for each dependent variable.

	**Model formulation**	**AIC**
Engagement	behavior1 ~ treatment * age+ (1∣ day period)	116.8
	behavior1 ~ treatment + age+ (1∣ day period)	**115.3**
Mount signals	behavior2 ~ treatment * age+ Mount signals + (1∣ day period)	81.5
	behavior2 ~ treatment + age+ Mount signals + (1∣ day period)	**79.0**
